# Targeted next-generation sequencing of *Mycobacterium tuberculosis* from patient samples: lessons learned from high drug-resistant burden clinical settings in Bangladesh

**DOI:** 10.1080/22221751.2024.2392656

**Published:** 2024-08-13

**Authors:** Mohammad Khaja Mafij Uddin, Andrea Maurizio Cabibbe, Rumana Nasrin, Arash Ghodousi, Fahim Alam Nobel, S. M. Mazidur Rahman, Shahriar Ahmed, Md. Fahim Ather, S. M. Abdur Razzaque, Md. Abu Raihan, Pronab Kumar Modak, Jean Luc Berland, Wayne Van Gemert, Sardar Munim Ibna Mohsin, Daniela Maria Cirillo, Sayera Banu

**Affiliations:** a Infectious Diseases Division, icddr,b, Dhaka, Bangladesh; b Emerging Bacterial Pathogens Unit, IRCCS San Raffaele Scientific Institute, Milan, Italy; c National Institute of Disease of the Chest and Hospital, Dhaka, Bangladesh; d 250 bed TB Hospital, Dhaka, Bangladesh; e National Tuberculosis Control Programme, Bangladesh; f Fondation Merieux, Lyon, France; g Stop TB Partnership, Switzerland; h Office of Population, Health, and Nutrition, U.S. Agency for International Development (USAID), Bangladesh

**Keywords:** Targeted next-generation sequencing, phenotypic drug susceptibility testing, mutation, diagnostic performance, lineage, feasibility, drug-resistant TB

## Abstract

Lack of appropriate early diagnostic tools for drug-resistant tuberculosis (DR-TB) and their incomplete drug susceptibility testing (DST) profiling is concerning for TB disease control. Existing methods, such as phenotypic DST (pDST), are time-consuming, while Xpert MTB/RIF (Xpert) and line probe assay (LPA) are limited to detecting resistance to few drugs. Targeted next-generation sequencing (tNGS) has been recently approved by WHO as an alternative approach for rapid and comprehensive DST. We aimed to investigate the performance and feasibility of tNGS for detecting DR-TB directly from clinical samples in Bangladesh. pDST, LPA and tNGS were performed among 264 sputum samples, either rifampicin-resistant (RR) or rifampicin-sensitive (RS) TB cases confirmed by Xpert assay. Resistotypes of tNGS were compared with pDST, LPA and composite reference standard (CRS, resistant if either pDST or LPA showed a resistant result). tNGS results revealed higher sensitivities for rifampicin (RIF) (99.3%), isoniazid (INH) (96.3%), fluoroquinolones (FQs) (94.4%), and aminoglycosides (AMGs) (100%) but comparatively lower for ethambutol (76.6%), streptomycin (68.7%), ethionamide (56.0%) and pyrazinamide (50.7%) when compared with pDST. The sensitivities of tNGS for INH, RIF, FQs and AMGs were 93.0%, 96.6%, 90.9%, and 100%, respectively and the specificities ranged from 91.3 to 100% when compared with CRS. This proof of concept study, conducted in a high-burden setting demonstrated that tNGS is a valuable tool for identifying DR-TB directly from the clinical specimens. Its feasibility in our laboratory suggests potential implementation and moving tNGS from research settings into clinical settings.

## Introduction

Multidrug-resistant or rifampicin-resistant tuberculosis (MDR/RR-TB) and extensively drug-resistant tuberculosis (XDR-TB) pose a significant public health concern [1]. Patients with RR or MDR-TB (resistant to at least rifampicin and isoniazid) have low treatment success rates and only one-third of people in need access to such treatments [2]. This is due to the lack of appropriate early diagnostic tools for drug-resistant TB (DR-TB) and their incomplete drug susceptibility testing (DST) profiling, which may lead to treatment failure and the emergence and spread of MDR, pre-XDR (defined as MDR-TB with additional resistance to fluoroquinolones) and XDR-TB (fulfil the definition of pre-XDR with additional resistance to bedaquiline or linezolid) isolates. The World Health Organization (WHO) endorsement of the novel BPaLM/BPaL regimen for people with MDR/RR-TB, presents a new diagnostic challenge under routine conditions. The current WHO-recommended rapid diagnostics are limited in targeting the number of drugs and genetic regions conferring resistance. They do not encompass most of the drugs present in the new regimens, at least within a single test [3].

The diagnostic challenges in detecting DR-TB can be addressed through whole-genome sequencing (WGS) or the recently introduced targeted next-generation sequencing (tNGS). While WGS is highly effective in detecting DR-TB, its reliance on primary culture leads to treatment delays [4–6]. It is also expensive and requires bioinformatics infrastructure and expertise to analyse the data. In contrast, tNGS has become an alternative tool that provides sequence information across a wide range of loci compared to existing molecular methods and delivering faster results than phenotypic DST (pDST) [7,8]. Recently, the WHO released a rapid communication advocating for the use of tNGS in diagnosing DR-TB, affirming its accuracy for prioritized patient populations needing comprehensive DST. They also highlighted its cost-effectiveness depending on the context and its feasibility for implementation under routine conditions [9]. WHO also endorsed tNGS products for comprehensive DR-TB detection directly from clinical specimens. The Deeplex Myc-TB assay is one of them. The Deeplex Myc-TB kit (GenoScreen, Lille, France) a commercial solution evaluated by the WHO, has met the class-based performance criteria for resistance testing of rifampicin (RIF), isoniazid (INH), pyrazinamide (PZA), ethambutol (EMB), fluoroquinolones (FQs), bedaquiline (BDQ), linezolid (LZN), clofazimine (CFZ), amikacin (AMK), kanamycin (KAN), capreomycin (CAP), ethionamide (ETH) and streptomycin (STR). This product has already been applied in a few countries for DR-TB detection and surveillance [10–12].

Bangladesh, a country with a high burden of TB and MDR/RR-TB, had an estimated 379,000 cases of TB in 2022, resulting in 42,000 deaths. The incidence rate of MDR/RR-TB is 2.9 (per 100,000 population) [2]. There are substantial gaps and challenges in the diagnosis of DR-TB in the country. Less than half of new cases of TB were tested for RIF in 2022, and coverage for FQs resistance testing for MDR/RR-TB cases is even lower, which is worrisome for setting a high burden of TB [13]. Successful diagnosis and selection of effective treatment of DR-TB depend on the rapid and accurate DST. In Bangladesh, WHO-approved pDST, line probe assay (LPA), both first and second line, and Xpert MTB/RIF (Xpert) assay are currently being used for DR-TB diagnosis. The country is currently implementing the GeneXpert XDR test in limited areas, which promises rapid and targeted detection of resistance to isoniazid, fluoroquinolones, ethionamide, and amikacin among TB patients. In addition to GeneXpert XDR (Xpert XDR), second-line LPA, which can also detect fluoroquinolones and aminoglycosides resistance patterns*.* However, alongside these, leveraging tNGS can offer additional benefits by providing comprehensive genetic data, resistance to newly introduced drugs (BDQ, LZN, CFZ), and enabling tailored treatment strategies, even in the presence of the Xpert XDR and LPA.

Our study aimed to provide proof of the principle of the performance and feasibility of tNGS in TB diagnostic algorithms in Bangladesh. We conducted a comparative analysis between tNGS using the Deeplex Myc-TB kit and pDST, Xpert, and LPA for direct DR-TB detection.

## Methods and materials

### Study population, sample size and sample collection

Between January and December 2022, participants were enrolled from three clinical sites: icddr,b TB Screening and Treatment Centres (TBSTCs), National Institute of Diseases of the Chest and Hospital (NIDCH), and 250 bed Shyamoli TB Hospital. The two hospitals are tertiary referral centers for TB and chest diseases in Bangladesh, situated in the central area of Dhaka Metropolitan City. Patients from across the country, often referred cases, seek treatment at these healthcare facilities. TBSTCs, located in Dhaka, operate as a sustainable Social Enterprise Model (SEM) for TB care within the private sector of Bangladesh. Approximately 40–50 MDR/RR TB patients visit these hospitals every month. Each Xpert positive pulmonary TB (PTB) patients, either rifampicin-resistant (RR) or sensitive (RS), aged more than 10 years and who have not yet started the anti-TB treatment were enrolled. During the study period, a total of 370 PTB (RR-270 and RS-100) patients were enrolled. In this evaluation study, more RR-TB cases than RS-TB were enrolled to better understand the mutational profiles that lead to resistance against first and second-line drugs, Due to a higher number of RS cases in these clinical sites, enrolment for this group was completed earlier compared to RR cases. After enrolment, a fresh sputum sample was collected from each participant and sent to the Mycobacteriology Laboratory of icddr,b where all phenotypic and genotypic DST were performed. The study protocol was approved by the Institutional Research Review Committee (RRC) and Ethical Review Committee (ERC) of icddr,b. Before enrolment, informed written consent was taken from each of the participants.

### Xpert MTB/RIF assay and culture

Xpert assay has emerged as an effective tool for the simultaneous detection of *Mycobacterium tuberculosis* complex (MTBC) and based on the five molecular beacon probes covering the high-prevalence mutations (i.e. “hot spots”) associated with RIF resistance. The Xpert assay was performed following manufacturer instructions [14]. The initial Xpert assay was performed at the clinical sites and a repeat Xpert test was performed on fresh sputum samples at the Mycobacterology laboratory. The GeneXpert System (Ver.4.0) with in-built software (Ver.6.0) was used for testing. The remaining sputum specimen was decontaminated and concentrated following modified Petroff’s method. In brief, the sputum sample was digested and decontaminated with an equal volume of 1% N-acetyl-L-cysteine (NALC), 4% NaOH, and 2.9% sodium citrate [15]. After centrifugation, the pellet was inoculated into two L-J slants and incubated at 37°C for 6–8 weeks. The remaining pellet was stored at – 20°C for LPA and tNGS.

### Phenotypic drug susceptibility testing

pDST was performed by L-J proportion method and MGIT 960 system (Becton Dickinson Diagnostic system, USA) using the WHO-recommended critical concentrations of the individual drugs mentioned in Supplementary Table 1. For the new and repurposed drugs, BDQ, CFZ, LZN, and PZA, pDST was performed with the MGIT 960 according to the manufacturer’s instructions [16]. For STR, INH, RIF, EMB, AMK, KAN, CAP, ETH, and FQs including LEV, Moxifloxacin (MOX), and Ofloxacin (OFL), pDST was performed on L-J media as described previously [17].

### Line probe assay

One portion of the specimen was directly subjected to the first- and second-line LPA (Genotype MTB DR*plus* Ver. 2.0 and MTBDR*sl* Ver 2.0; Hain Lifescience, Germany) as per the manufacturer’s guidelines. The first line LPA was used to detect resistance to RIF and INH [4], whereas the second line was used for FQs and aminoglycosides (AMG) [5].

### DNA extraction, target amplification and next generation sequencing

We used Deeplex Myc-TB kit for tNGS directly from the clinical specimens. After inoculation on culture, the remaining pellets were heat-inactivated at 95°C for 30 min, digested with proteinase K (Qiagen, Germany) and Lysozyme (Sigma, St. Louis, Missouri, United States) for at least 4 h at 37°C and DNA was then extracted using Genomic-tips DNA extraction kit (Qiagen, Germany). After DNA extraction, PCR amplification of targeted regions was performed using Deeplex Myc-TB kit according to the manufacturer’s instructions. The amplicons were cleaned with AMPure XP magnetic beads (Beckman Coulter, CA, USA) and purified products were then quantified with Qubit dsDNA HS Assay kit (Life Technologies, UK). The Illumina DNA Library Prep kit (Illumina Inc., San Diego, USA) was used to obtain paired-end libraries. The sequencing was performed with Illumina MiniSeq platform using the Mid Output kit (300 cycles) which can sequence 24 samples. A cloud-based web tool (Deeplex Myc-TB, Ver. 3.0) provided by GenoScreen was used to extrapolate the susceptibility pattern from the sequence. The sequence qualities were categorized into +++ (where all resistance-associated positions with enough data to identify mutations from 3 to 100%), ++ (10–100%), + (80–100%) – (one or more resistance-associated positions not covered) and MTB not detected. External quality assessment (EQA) was performed to check the quality of protocol and sequencing results. An EQA panel comprising of ten well characterized isolates was received from the WHO Collaborating Centre in Tuberculosis Laboratory Strengthening (ITA-98) and WHO/IUATLD Supranational Reference Laboratory (SRL), Milan, Italy. Sequencing was performed among these isolates according to the above-mentioned protocol. Additionally, 10% of randomly selected samples were sent to the SRL for retesting. Both the EQA testing results were aligned with the original results of each site.

### Feasibility component and outcome measure

The feasibility of introducing tNGS in our laboratory involved two key phases; the preparatory and implementation phases of the assay. During the preparatory phase, we focused on procuring reagents and equipment, establishing laboratory infrastructure, recruiting personnel, and providing comprehensive training on the tNGS assay and data analysis. The implementation phase included ensuring specimen flow, executing the tNGS workflow, conducting data analysis, and reporting results. The turnaround time for the entire process was also considered during this phase. The study's outcome measures included successful implementation and the establishment of an accessible and sustainable tool for detecting DR-TB in our setting.

### Statistical analysis

All statistical analyses were conducted using STATA (version 20.0) and R (version 4.3.1) software packages. We used descriptive statistics, including mean and median, to summarize the socio-demographic characteristics such as age and gender. The proportions of isolates belonging to each lineage were also calculated. We assessed the performance of tNGS compared to pDST and LPA separately, and with a composite reference standard (CRS) consisting of both tests to calculate sensitivity and specificity with 95% confidence intervals (CI). In the CRS, isolates were considered resistant if either test (pDST or LPA) showed a resistant result.

## Results

### Socio-demographic and clinical characteristics of patients

**Figure 1.**
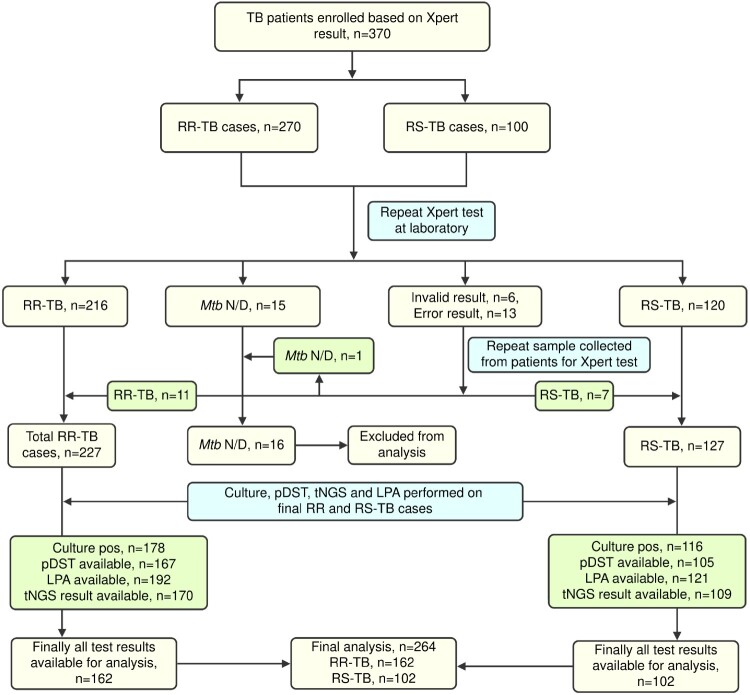
Study flow chart including the overall study outline and result summary of repeat Xpert, pDST, LPA and tNGS. TB patients either RR or RS determined by Xpert assay at the clinical sites were enrolled. Repeat Xpert, pDST, LPA, and tNGS were performed on fresh sputum at the central Mycobacteriology laboratory. RR: Rifampicin resistant; RS: Rifampicin sensitive; N/D: not detected.

During the study period, we enrolled 270 RR and 100 RS-TB patients based on the initial Xpert test at the clinical sites. Of 370 collected specimens, 227 were RR, 127 RS, and the remaining 16 cases were negative for MTBC on repeat Xpert testing. Any invalid or error results were repeated with fresh sputum samples for final confirmation. Out of 227 RR and 127 RS-TB cases, 294 were culture-positive, of which pDST results were available for 272 isolates. LPA and tNGS results were available for 313 and 279 isolates. Considering the availability of pDST, LPA, and tNGS results, we had 264 specimens for analysis, of which 162 (61.3%) were RR, and 102 (38.6%) were RS determined by repeat Xpert assay (Figure 1).

The median age of the patients was 34 years (Interquartile range [IQR] 24–48); 62.5% were male and 71.2% of the patients had no previous history of TB. tNGS results showed that among the RS cases, 79 (77.5%) patients were sensitive to all anti-TB drugs, and ten (9.8%) were mono-resistant to STR, INH, and other resistance to ETH, or FQs were seven (6.9%) in tNGS. Two cases were found as MDR-TB among RS cases. Conversely, among RR cases, eight (4.9%) patients identified as RR in initial Xpert but deemed RS in the repeat Xpert test, were found as sensitive to all anti-TB drugs. A total of 98 (60.5%) were MDR-TB, and 26 (16.0%) were detected as pre-XDR-TB according to the 2021 WHO definition (Table 1). The higher rate of MDR/pre-XDR-TB is not reflective of the country, as the higher number of RR-TB cases than RS-TB cases were enrolled to better understand more about the mutations that confer resistance to the second-line, new and repurposed drugs. Out of the 102 cases diagnosed as RS by Xpert, two cases were identified as RR with tNGS. Among the two isolates, one exhibited a mutation at H445N with an allelic frequency of 59.7% in tNGS, which was not common in the Xpert assay. In another isolate, the mutation at S450L (allelic frequency of 82.2%), commonly associated with rifampicin resistance, was detected in tNGS despite no mutation being identified by Xpert. Conversely, among the 164 cases diagnosed as RR by Xpert, ten cases were found to be RS in the tNGS assay. Furthermore, out of ten isolates demonstrating RR in the Xpert assay, eight had low/very burden with no probe failure and/or ΔCt value > 4.0 and the remaining two isolates had specific probe failure (probe E). In tNGS, one isolate had the in-frame *rpoB* deletion, another isolate had low frequency *rpoB* mutation and no mutations were found for the remaining eight isolates, and hence these were considered as RS.

**Table 1.** Socio-demographic and clinical characteristics (a), and drug-resistant profiles with tNGS of enrolled patients (*n* = 264) according to Xpert status (b).

a) Demographic Characteristics		Number of patients	Frequency (%)
Sex	Male	165	62.5
	Female	99	37.5
Age ranges	0–20	45	17.0
	21–35	105	39.8
	36–50	64	24.3
	>50	50	18.9
TB History	Yes	76	28.8
	No	188	71.2
b) Resistant profile		RS (*n* = 102, %)	RR (*n* = 162, %)
	All Sensitive	79 (77.5)	8 (4.9)
	Mono-resistant^§^		
	STR INH RIF Other resistant ETH FQs	8 (7.8) 2 (1.9) – 2 (1.9) 5 (4.9)	– – 24 (14.8) – 1 (0.6)
	Polyresistance*	4 (3.9)	5 (3.0)
	MDR-TB^#^	2 (1.9)	98 (60.5)
	Pre-XDR^ᵠ^	–	26 (16)

^§^ (resistance to one first-line anti-TB drug only), *(resistant other than RIF + INH + FQ + AMG), # (INH + RIF with any other drugs), ᵠ(MDR-TB/RR-TB + FQ).

### Drug resistance detection in different tests

**Figure 2.**
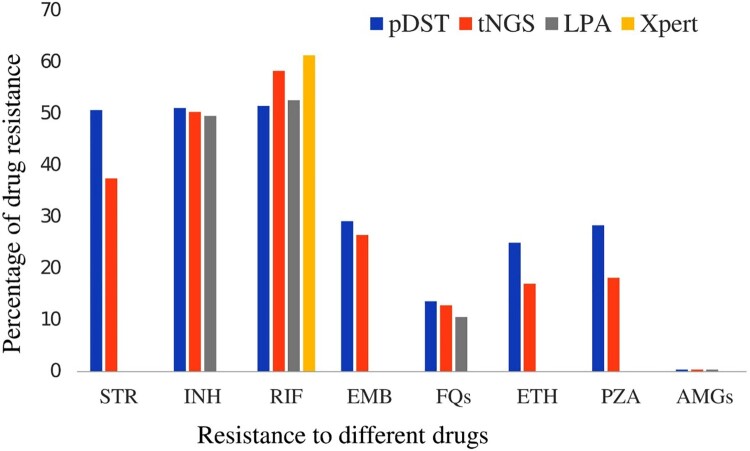
Comparison of different drug susceptibility testing (pDST, tNGS, LPA, and Xpert) methods for drug resistance detection.

The data presented in Figure 2 compares the frequencies of drug resistance using four different tests: pDST, tNGS, LPA and Xpert assays separately. The results indicate that pDST had the highest resistant frequencies among all drugs except RIF. On the other hand, tNGS and Xpert showed higher resistance frequencies for RIF than other tests. Only one isolate was found to have resistance to AMGs, and there were no differences in resistance determination using any of the methods. All isolates were found to be susceptible to BDQ, LZN and CFZ using either pDST or tNGS (Figure 2).

### Comparison of tNGS sequence quality with Xpert semi-quantitative assay

**Figure 3.**
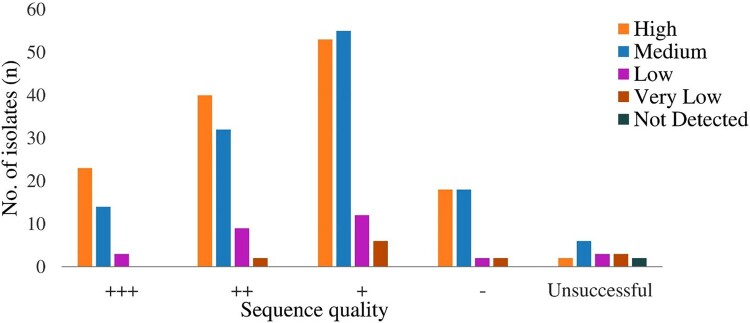
Sequencing quality obtained by Deeplex-Myc TB assay stratified with Xpert semi-quantitative burden (High, Medium, Low, and Very Low).

tNGS was performed on 305 samples. Of these, 289 (94.8%) were considered successful with varying sequence quality, while 16 (5.2%) were unsuccessful. A total of 98.5% (134/136) of high burden, 95.2% (119/125) of medium burden, 89.6% (26/29) of low burden and 76.9% (10/13) of very low burden load samples determined by Xpert have produced results, respectively. The study also found high-quality sequences (ranging from + to +++) among samples ranging from high to low bacterial burden in the Xpert assay compared with samples with very low bacterial burden (Figure 3).

### Performance of tNGS directly from clinical specimens compared to pDST

The diagnostic performance of tNGS was evaluated compared to pDST as a reference standard (Table 2). Sensitivity and specificity were calculated compared to pDST. The accuracy of tNGS in predicting resistant phenotypes detected by pDST was >93% for most key drugs (INH, RIF, FQs, and AMGs). The sensitivities were 68.7%, 96.3%, 99.3%, and 76.6%, and specificities were 94.6%, 97.7%, 85.1%, and 94.1% for STR, INH, RIF and EMB respectively. On the other hand, the sensitivities were 94.4 and 100% for FQs and AMGs respectively. The specificities were 100% for each of these drugs. Lower sensitivities of 56.0% and 50.7% were found for ETH and PZA respectively.

**Table 2.** Diagnostic performance evaluation of tNGS by drugs compared to pDST as the reference standard (*n* = 264).

	pDST Resistance			pDST Susceptible			Sensitivity (95% CI)	Specificity (95% CI)
	tNGS			tNGS				
Drugs	R	S	Total	R	S	Total		
STR	92	42	134	7	123	130	68.7 (60.0–76.4)	94.6 (89.2–97.8)
INH	130	5	135	3	126	129	96.3 (91.6–98.8)	97.7 (93.4–99.5)
RIF	135	1	136	19	109	128	99.3 (95.8–99.9)	85.2 (77.8–90.8)
EMB	59	18	77	11	176	187	76.6 (65.6–85.5)	94.1 (89.7–97)
FQs	34	2	36	0	228	228	94.4 (81.3–99.3)	100 (98.4–100)
AMGs	1	0	1	0	263	263	100 (2.5–100)	100 (98.6–100)
ETH	37	29	66	8	190	198	56.0 (43.3–68.3)	95.9 (92.1–98.2)
PZA	38	37	75	10	179	189	50.7 (38.9–62.4)	94.7 (90.4–97.4)

R = resistant, S = sensitive.

Of the 659 resistant phenotypes with pDST, 525 (79.7%) were also showed resistant phenotypes and the remaining 134 (20.3%) were predicted as susceptible phenotypes with tNGS. These discrepancies were mostly obtained among STR (41 isolates), PZA (37 isolates), ETH (30 isolates), and EMB (18 isolates). In pDST, 41 STR resistant phenotypes were classified as sensitive in tNGS due to the absence of any resistance-associated mutations, or the presence of uncharacterized mutations, or insufficient coverage of the STR target genes of *gidB* and *rpsL*. Among these cases, 28 exhibited wild-type, while seven displayed uncharacterized mutations, three isolates with low coverage, and three cases harboured minority variants (P84L, K43R, and K88R) within the STR target genes. In the pDST results, out of 37 PZA resistance phenotypes, 27 isolates exhibited wild-type phenotype, one isolate showed an uncharacterized mutation, five had insufficient coverage of the *pncA* target gene, and four had minority variants identified as A46 V, Q10H, P54L, and G97S. Regarding EMB resistance, among 18 phenotypes, 11 isolates displayed the wild-type phenotype, three had low coverage, one exhibited an uncharacterized mutation in the *embB* target gene, and two had minority variants identified as M306I and G406S, while one isolate had a mutation at G406D with an allelic frequency of 98%. Among the 30 ETH-resistant phenotypes, 17 isolates had no mutations detected, three had low coverage, and ten isolates showed uncharacterized mutations at the *ethA, fabG1*, and *inhA* target genes.

Among the five INH susceptible isolates in tNGS but resistant in pDST, three (60%) had uncharacterized mutations at g-142a of the INH target gene of *ahpC*, and two had no mutations at any of the INH target genes. One isolate was found RR in pDST but susceptible in tNGS due to the uncharacterized mutation of delCAG (deletion) at 761112-4 genomic position of *rpoB* gene, but according to the new version of the WHO catalog, in-frame deletions in *rpoB* hotspot are considered markers of resistance. Among two phenotypically FQs resistant isolates, one had a minority variant of A90 V (frequency of 3.0%) and the other isolate had good quality sequence (depth coverage of 272 for *gyrA* and 334 for *gyrB*) with wild-type pattern in tNGS. However, this discrepancy is likely due to the suboptimal sensitivity of tNGS.

Of the 1978 susceptible phenotypes in pDST, 58 (2.9%) were discordantly predicted as resistant in tNGS. Discordant results (19/58; 32.7%) were mostly noticed in RIF due to the disputed mutations. Among 19 RIF susceptible in pDST but resistant in tNGS, nine had a mutation at L430P; four had H445N, two had L452P mutations and S450W, N437Y, H445Q, and D435Y mutations for one each isolate (Supplementary Table 2). Of three INH susceptible isolates in pDST, two had common mutations at S315 T, and another one had the same mutation as a minor variant (allelic frequency of 41.2%). The former two isolates were also identified as INH resistant in LPA, where wild-type probes were absent and MUT1 (Mutant) probes were detected. Among seven STR susceptible cases in pDST, one had a common mutation at K43R and six had mutations at L74*, Y195*E99*, A138 T, and delC/G (frameshift) of STR target genes. Phenotypically sensitive but resistant in tNGS were also found among EMB for 11 isolates, eight for ETH and ten for PZA.

### Comparison of tNGS results with LPA directly from sputum specimens

The sensitivities and specificities of tNGS for INH, RIF, FQs and AMG were 96.5%, 96.4%, 96.6%, 100% and 96.9%, 84.0%, 97.9% and 100%, respectively, when compared to LPA (Table 3). Additionally, 20 isolates were observed as RR in tNGS but susceptible in LPA due to the minority variants in *rpoB*: L430P, H445N/D/R, D435D/V, L452P, and S450L. Four isolates were INH-resistant in tNGS but susceptible in LPA, of which one showed mutation at *fabG1* gene (c-15t) and the other three had mutation at S315 T in the *katG* gene. Of five FQs resistant cases, two were due to the rare mutation of E501D in the *gyrB* gene, whereas three were due to the mutation at D89N, A90 V, and D94A in *gyrA* gene.

**Table 3.** Sensitivity and specificity of tNGS compared to LPA as the reference standard directly from clinical specimens (*n* = 264).

	LPA Resistance			LPA Susceptible			Sensitivity (95% CI)	Specificity (95% CI)
	tNGS			tNGS				
Drug name	R	S	Total	R	S	Total		
INH	127	6	133	4	124	128	96.5 (90.4–98.3)	96.9 (92.2–99.1)
RIF	134	5	139	20	105	125	96.4 (91.8–98.8)	84.0 (76.4–89.9)
FQs	28	1	29	5	230	235	96.6 (82.2–99.9)	97.9 (95.1–99.3)
AMG	1	0	1	0	263	263	100 (2.5–100.0)	100.0 (98.6–100.0)

R = resistant, S = sensitive.

The mutation frequencies of different gene targets for most key drugs were also analysed. The most frequent variant found in *rpoB* was S450L (79 isolates, 51.2%), followed by H445D or other substitutions (18.9%, 29 isolates). The most prevalent variant associated with INH resistance phenotype was S315 T (78.2%; 104 isolates) of the *katG* gene. For FQs, in the *gyrA* gene, the most frequent mutation was found with codons D94G/A/Y/N (45.5%; 15 isolates) and A90 V (36.4%, 12 isolates). Resistance to AMGs was found only for 1 isolate with codon a1401 g of *rrs1* gene. The distribution of frequency mutations among gene targets of different drugs is shown in Supplementary Table 3.

### Diagnostic performance of tNGS compared to composite reference standard (CRS)

To better understand the performance of tNGS, the results of tNGS were compared with the CRS. Compared to CRS, where the reference is considered as resistant if resistant in either LPA or pDST, the sensitivities of tNGS for INH, RIF, FQs and AMGs were 93.0%, 96.6%, 90.9%, and 100%, respectively. The specificities ranged from 91.3% to 100% for the above key drugs (Table 4).

**Table 4.** Diagnostic performances of tNGS compared to composite reference standard (CRS).

	CRS Resistance			CRS Susceptible			Sensitivity (95% CI)	Specificity (95% CI)
	tNGS			tNGS				
Drug name	R	S	Total	R	S	Total		
INH	133	10	143	0	121	121	93.0 (87.5–96.6)	100 (97.0–100)
RIF	144	5	149	10	105	115	96.6 (92.3–98.9)	91.3 (84.6–95.7)
FQs	30	3	33	3	228	231	90.9 (75.7–98.0)	98.7(96.2–99.7)
AMGs	1	0	1	0	263	263	100 (2.5–100)	100.0 (98.6–100)

R = resistant, S = sensitive.

### Distribution of SNP-based lineages and their association with drug susceptibility patterns

Four different lineages: Lineage 1 (EAI), Lineage 2 (Beijing), Lineage 3 (Delhi/CAS) and Lineage 4 (EAS/LAM), were determined based on single nucleotide polymorphism (SNP) detected by tNGS. The lineages were also divided into different sub-lineages. About 32.9% (87) of the isolates were identified within lineage 1, sub-lineages 1.1.2, and 1.2.2 followed by lineage 4, other than 4.9, and sub-lineages 4.3 (72; 27.3%); lineage 2 and lineages 2.2.1 (67; 25.4%) and lineage 3 and lineage 3.1.1 (38; 14.4%). In all susceptible isolates, lineage 1 and lineage 3 (25.3 and 34.2%) were more represented than other lineages. In contrast, lineage 2 and lineage 4 (Beijing lineage) were more predominant among any drug resistant (82.1 and 83.3%) or MDR/pre-XDR-TB cases (52.2 and 54.2%) (Table 5).

**Table 5.** Lineage distribution of MTBC isolates among the enrolled patients (*n* = 264).

Lineage	Number	%	All Susceptible (59)	%	Any drug-resistant (205)	%	MDR-TB/Pre-XDR (109)	%
Lineage 1 (EAI) Lineage 1.1.2 Lineage 1.2.2	63 6 18	32.9	16 0 6	25.3	47 6 12	74.7	24 2 4	34.5
Lineage 2 (Beijing) Lineage 2.2.1	64 3	25.4	12 0	17.9	52 3	82.1	33 2	52.2
Lineage 3 (Delhi/CAS) Lineage 3.1.1	12 26	14.4	6 7	34.2	6 19	65.8	5 10	39.5
Lineage 4 (EAS/LAM) Lineage 4.3 Lineage other than 4.9	14 3 55	27.3	2 2 8	16.7	12 1 47	83.3	9 1 29	54.2

### Feasibility of the use of tNGS in the laboratory setting

The feasibility of implementing tNGS in the laboratory setting comprised two crucial phases: preparation and implementation. The preparatory phase encompassed procurement, laboratory setup, and training. Procuring different reagents and equipment proved to be highly challenging due to unavailability of distributors within the country. Laboratory infrastructure including adequate space, benchtop, power supply, internet connectivity and required temperature were ensured during the renovation of the sequencing facility. Personnel were trained on tNGS workflow, equipment handling and data analysis. The comprehensive training included theoretical and hands-on sessions were conducted by team members from the Milan SRL and GenoScreen. The implementation phase focused on the practical work of tNGS with study samples. Sequencing data were analysed with the cloud-based Deeplex Web application tool. The turnaround time, starting from DNA extraction to sequencing data analysis, was 3–4 days. We successfully set up and implemented this technology for DR-TB detection directly from clinical specimens in our laboratory setting. The approximate cost of tNGS per sample was $ 150, considering the cost of reagents, other consumables and human resources (HR, 10% of the total cost). The median cost for conventional DST for all drugs is around $140, almost the same as that of tNGS. With an increase in sample volume and the use of high-output kit that covers more samples in each run, the cost of tNGS is expected to decrease compared to the current pricing.

## Discussion

Our study findings demonstrated that tNGS exhibits excellent performance compared to pDST and can be used as a valuable tool for rapidly identifying DR-TB directly from clinical specimens. To reach the targets of the End TB strategy by 2030, DST must be performed to detect DR-TB for each single confirmed TB patient. Currently, there is a lack of WHO-recommended rapid diagnostics that can comprehensively detect resistance to all drugs of recently guided regimens in a single test [9].

pDST requires extended time, from weeks to months of incubation, and often lacks accuracy, reproducibility, especially for drugs such as pyrazinamide [18] or newly introduced BDQ/LZN/CFZ/ pretomanid [7]. For these reasons, the adoption of rapid molecular tests has been shown to lower the time to treatment initiation in patients with TB and DR-TB. Current tests (Xpert and LPA) cannot be adapted easily to detect new genomic regions associated with resistance to novel and repurposed TB medications recently approved for treatment by WHO [19]. In this study, we aimed to investigate using Deeplex Myc-TB to identify the DR-TB directly from clinical samples. We compared tNGS results with the Xpert, pDST and LPA. According to the country data for 2022, the estimated rates of RR/MDR-TB among new and previously treated TB patients are 1.1 and 5.5%, respectively. Considering the patient enrolment criteria, a high proportion of Xpert-confirmed RR-TB patients were enrolled, and hence, in tNGS a high ratio of RR/MDR-TB (124/264; 46.9%) was found among the enrolled patients. Similarly, a high rate of pre-XDR-TB (26/264; 9.8%) was also found. These figures do not reflect the actual MDR-TB and pre-XDR burden in the country: the enrolment of more RR cases in this study was done to understand better the drug resistance patterns using tNGS as an initial implementation phase.

When we compared the resistance patterns with four different methods, the higher number of resistance patterns with pDST methods were mostly found for drugs other than INH, FQs, and AMGs. In a study conducted by Mansoor et al., tNGS was associated with higher resistance compared to pDST, with higher mutations associated with drugs causing MDR/pre-XDR/XDR-TB [8]. Resistance to new and repurposed drugs, such as BDQ, LZN, and CFZ, was absent in both tNGS and pDST due to resistance to these drugs not being common among RR-TB cases in the country. As the country has just initiated the implementation of the BPaL/BPaLM regimen, there has yet to be data on the rate of resistance to these drugs [20].

The sequence quality with good coverage of all targets depends on several factors, including the quality of samples, bacterial load in the specimens, efficient DNA extraction procedure, and DNA concentration after PCR. In our study, sequences were not detected or found to have low coverage with various gene targets for some samples. These phenomena mostly occurred with negative or very low bacterial burden specimens, consistent with the other studies [8,21]. It has been suggested that the sequence quality is highly influenced by bacterial burden. The size of the amplicons may vary, ranging from 0.5 to 1.0 (ng/µL) for a successful sequence.

Xpert testing with the same or new specimen can result in false positive RR cases (Xpert RR but confirmed as RS with other genotypic or phenotypic methods). Out of 264 cases, discrepancies between Xpert and tNGS were found for 12 isolates. Two isolates were initially classified as RS by Xpert but RR by tNGS. One of these isolates exhibited a mutation at H445N in tNGS. This isolate also found RS when tested with standard rifampicin critical concentration in pDST. It has been reported that the H445N mutation is linked to low levels of RIF resistance (with a minimum inhibitory concentration of 0.25). Moreover, this mutation is also known as a disputed mutation found both in resistant or susceptible isolates [22]. The other isolate was also found RR in pDST which was consistent with the tNGS result. Most of the putatively false RR cases (eight out of ten) were obtained from low and very low bacterial burden cases due to the no probe failure or delayed binding of the probes with the target sequence. In our previous study and also several other studies have reported false RR cases among low/very low bacterial samples. The discordant results can also be attributed to other factors including technical errors and sample quality and volume [23-25].

pDST using L-J DST was conducted for the majority of drugs, excluding PZA, LNZ, BDQ, and CFZ. Presently, the BACTEC MGIT 960 liquid culture method stands as the sole WHO-endorsed approach for testing the susceptibility of these drugs. Employing two distinct media may not affect the pDST outcomes [26]*.* Discordance between pDST and tNGS can lead to misinterpretation of results, which can affect appropriate treatment for patients. Among the resistant phenotypes in pDST, 20% were susceptible by tNGS. Most of the discrepancies were obtained among STR, PZA, EMB, and ETH. These discrepancies were primarily due to the uncharacterized or no mutations of different target genes. Out of 37 phenotypes showing resistance to PZA, 27 isolates exhibited no mutations in the *pncA* gene, suggesting that these resistance profiles may be attributed to alternative mechanisms such as mutations in efflux pump genes or other genes like *panD* and *rpsA*, which were not covered by tNGS using the Deeplex Myc-TB kit. Our previous study also reported discrepancies among phenotypic and sequencing results for PZA [27]. There were 17 isolates initially classified as ETH resistant in pDST despite lacking resistance-associated mutations. One isolate with the *embB* codon G406D mutation, which tested EMB sensitive in tNGS, should be considered EMB resistant regardless of pDST results. Two other mutations, *embB* codons G406S and M306I, were also linked to EMB resistance, but their presence as minority variants led to these isolates being identified as EMB sensitive in tNGS [28]. Similarly, three isolates harboured STR resistance-associated mutations specifically, K43R, P84L, and K88R at low frequencies (2–6%) in tNGS, indicating STR sensitive, yet they were deemed resistant in pDST. However, either pDST for a given drug is not reliable (e.g. false positive results) or tNGS sensitivity is sub-optimal. The discrepancies between tNGS and pDST for these drugs result in significant variations in the sensitivity of tNGS assays across different studies [29].

Among resistant phenotypes, susceptibility to INH, RIF, and FQs in tNGS had occurred within very few isolates, mostly due to uncharacterized or no mutation of specific target genes. Resistance in pDST may be influenced by factors other than genetic mutations, including variations in growth conditions. Moreover, mutations are unknown or incomplete for certain drugs in tNGS. Conversely, among the susceptible phenotypes with pDST, only 2.9% showed resistance by tNGS. The discrepancy was more frequently found in RIF (32.7%). This RR occurred due to the disputed mutations in the *rpoB* gene, mostly found in L430P, H445N, D435Y, L452P, and S450W/L. The obtained disputed mutations for the low level of RR cases were consistent with the previously reported literature [25]. Some other countries, including Bangladesh, Kuwait, and South Korea have also reported higher frequencies of disputed mutations with variations among the populations [30,31]. Three isolates were displayed INH susceptible in pDST but resistant in tNGS. These discrepancies between tNGS resistance due to mutation at S315 T and pDST INH sensitive are not common. This might occur due to several factors, such as tNGS potentially identifying a small subpopulation with resistance-conferring mutations, the existence of both resistant and susceptible isolates within the same sample, and pDST may miss certain resistant subpopulations. Furthermore, errors during sample handling, variability in sample quality, and issues with sample volume could also contribute to these discrepancies. In the WHO catalog mutations associated with phenotypic resistance were validated through rigorous and standardized interpretation. Generally, the presence of a graded mutation would take precedence over the results of phenotypic testing. Conversely, the absence of graded mutations, particularly for second-line and new/repurposed drugs in RR/MDR isolates, would necessitate phenotypic confirmation*.* The discordance between tNGS and pDST has several implications for clinical practice. This may increase the risk of treatment failure and potentially foster the development of drug resistance. The clinicians should ensure a comprehensive assessment of drug resistance, utilizing both genetic and phenotypic data to guide treatment decisions effectively.

We also found discrepancies in resistance detection between tNGS and LPA (both 1st and 2nd line). Specifically, discrepancies arose in 20 isolates concerning RIF resistance: tNGS detected RR while LPA showed RS. Among 20 isolates, six displayed mutations at H445D/N/R while seven had mutations at L452P, S450L, and D435Y/V. Another seven isolates revealed mutations at L430P/R and D435F which were missed by LPA and these mutations are rare and only detected theoretically (*in silico*) in LPA. Discrepancies were also noted in INH resistance detection for four isolates, one of which showed INH resistance due to a mutation at c-15t of the *fabG1* gene, which was absent in LPA. The remaining three had a common mutation at S315 T of *katG* gene which was undetected in LPA. Among the five isolates showing FQ resistance in tNGS but sensitive in LPA, two were attributed to mutations in the E501D of the *gyrB* gene and one due to the D89N mutation in the *gyrA* gene, which were not covered or at least no wild-type probe was present in second-line LPA. One isolate had a common mutation at A90 V and another one had a minority variant of D94A (frequency of 21%) with the *gyrA* gene, which was missed in LPA [32,33].

In this study, 78.2% of mutations that confer resistance to INH were found at S315 T of *katG* gene, and 12.8% of mutations were found at c-15t of *fabG1* gene. In a study conducted by Welekidan, 78% of INH resistance was found among the S315 T codon of the *katG*, which is very similar to our findings [34]. Another previous study reported 96.5% resistance in the same codon [35]. In this study, the most frequent mutation was noticed in S450L (54.6%) of the *rpoB* gene among the RR-TB cases. The current study finding is similar to previous studies from Taiwan (49.4%) and India (57.8%) [36,37]. In our study, the frequency of mutations at codon S450L is higher than in one study conducted in Vietnam (37.8%) [38]. On the other hand, the frequency of mutation at the same codon was lower than in previous studies from different regions of Ethiopia (70%−80%) [34,39]. For FQs resistant cases, the most frequent mutations were found at *gyrA* codon D94G (45.5%) and A90 V (36.4%), which is very similar to our previous study and other studies where the most frequent mutations were found in the *gyrA* gene [40,41]. Resistance to AMG (KAN, AMK, CAP) was detected only in one isolate at *rrs* gene of codon A1401G, which aligns with the previously reported study [35].

Studies have reported that Lineage 2 (Beijing) isolates are highly associated with Pre-XDR and XDR-TB, while other studies have reported high rates of sensitive or drug-resistant (except MDR/Pre-XDR/XDR-TB) isolates in association with lineage 1 (EAI) [42,43]. In this study, Lineage 2, Lineage 4 and their sub-lineages were mostly observed in MDR/Pre-XDR cases whereas Lineage 1 was distributed with the drug susceptible isolates, which is consistent with a previous study conducted in Bangladesh [43].

Based on our experiences on the feasibility of the use of tNGS such as setting up laboratory infrastructures and other requirements and recent recommendation by the WHO, it is essential to move this technology from the research laboratory settings to National TB Reference Laboratory (NTRL) and subsequently to Regional TB Reference Laboratories (RTRLs) under the National TB Control Program where routine diagnostic tests are performed for detecting DR-TB. Careful planning, the initial selection of implementation sites, setting up laboratory infrastructures, ensuring an uninterrupted supply chain, and HR are the most critical parameters for the programmatic implementation of tNGS.

This study had several limitations. The sequence quality observed with the Deeplex Myc-TB kit was comparatively lower in samples having very low/low bacillary loads, potentially affecting the diagnostic performance of tNGS. Neither pDST nor tNGS detected resistance to new and repurposed drugs like BDQ, CFZ and LZN, which limits the mutational spectrum for all drugs in this study. The discrepancies were mainly obtained in PZA results from pDST using MGIT. According to the WHO recent recommendation, it has been found that PZA susceptibility with MGIT is associated with a high rate of false-positive resistance results. Moreover, inoculum preparation is important for conducting PZA testing reliably.

tNGS has several advantages and disadvantages. It can detect a broader range of genetic mutations associated with drug resistance compared to other rapid assays, and it has the potential for higher sensitivity. Although the initial cost of tNGS implementation may be higher than Xpert or LPA, the scalability of tNGS could lead to cost savings in the longer term. However, tNGS has a longer turnaround time for results compared to Xpert and LPA, and it requires sophisticated laboratory infrastructure and expertise, which may not be readily available in resource-limited settings. The Xpert XDR test can only identify resistance to specific drugs, while tNGS offers a complete drug resistance profile for targeted drugs. Implementation of tNGS could help to ensure quick and effective patient management. Establishing a standardized diagnostic algorithm is crucial, ensuring that individuals diagnosed with MDR/RR-TB via Xpert/TrueNat and treated with an all-oral MDR-TB regimen undergo tNGS testing. This process aims to detect mutations associated with drugs such as BDQ, FQs, LZN, and other key anti-TB drugs. Initially, tNGS can be implemented at the National TB Reference Laboratory, serving patients nationwide referred for DR-TB diagnosis. In integrating tNGS into the diagnostic algorithm alongside Xpert XDR and LPA, careful consideration should be given to factors such as costs, sample referral processes, turnaround times, and the range of drugs covered. tNGS has the potential to improve diagnostic precision through its comprehensive analysis, complementing the capabilities of GeneXpert XDR and LPA. Additionally, if the prevalence of resistance to drugs not covered by Xpert XDR/LPA increases over time, tNGS could prove to be a more cost-effective solution by reducing the need for additional diagnostic tests.

In our laboratory settings, tNGS provides added value to improve the detection of DR-TB for prioritized patient populations, in addition to existing WHO-approved molecular tests that are more accessible, cheaper and easier to perform for detecting resistance to most of the potential drugs. We found that the tNGS provided rapid results directly from the clinical specimens, reducing the turnaround time from 4–6 weeks for pDST to 3–4 days. We also found that tNGS had high sensitivity and specificity for most key drugs. tNGS can be an alternative diagnostic for patients requiring comprehensive pDST within a very short time or in areas where the facility for pDST is limited. This evaluation and feasibility study suggest that tNGS directly from the clinical specimens is feasible and will be useful for implementing and moving tNGS from research settings into clinical settings, e.g. initially at the NTRL and gradually at the RTRLs in the country.

## Supplementary Material

Supplementary_Table.docx
