# Supplementary material for: Targeted next-generation sequencing of Mycobacterium tuberculosis from patient samples: lessons learned from high drug-resistant burden clinical settings in Bangladesh
Source: Emerg Microbes Infect. 2024 Aug 13;13(1):2392656. doi: 10.1080/22221751.2024.2392656 (PMC11348811; doi:10.1080/22221751.2024.2392656)
Supplement: Supplementary_Table.docx [file TEMI_A_2392656_SM1493.docx]

**Supplementary Tables**

**Supplementary Table 1.** Target gene covered by Deeplex Myc-TB kit for drug resistance prediction and critical concentrations of drugs used for phenotypic DST

| **Drugs** | **Gene targets** | **Critical drug conc. (µg/mL)** |
| --- | --- | --- |
|  |  | **L-J DST** |
| RIF | *rpoB* | 40.0 |
| INH | *ahpC, fabG1, katG, inhA* | 0.2 |
| EMB | *embB* | 2.0 |
| SM | *gidB, rrs, rpsL* | 4.0 |
| OFL | *gyrA, gyrB* | 4.0 |
| LEV | *gyrA, gyrB* | 2.0 |
| MOX | *gyrA, gyrB* | 1.0 |
| ETH | *ethA, fabG1, inhA,* | 40.0 |
| AMK | *rrs* | 30.0 |
| KAN | *eis, rrs* | 30.0 |
| CAP | *rrs, tlyA* | 40.0 |
|  |  | **MGIT-AST** |
| PZA | *pncA* | 100.0 |
| LZN | *rplC, rrl* | 1.0 |
| BDQ | *Rv0678* | 1.0 |
| CFZ | *Rv0678* | 1.0 |

**Supplementary Table 2.** Disputed mutations in *rpoB* gene targets caused resistance to Rifampicin in tNGS

| Amino acid changes | Type of mutation | Number of isolates |
| --- | --- | --- |
| ctg430ccg | L430P | 9 |
| cac445aac | H445N | 4 |
| ctg452ccg | L452P | 2 |
| tcg450tgg | S450W | 1 |
| aac437gac | N437D | 1 |
| cac445caa | H445Q | 1 |
| gac435tac | D435Y | 1 |

**Supplementary Table 3.** Distribution and frequencies of mutations among different gene targets

| Drug | Gene target | Codon changes | Frequencies (%) |
| --- | --- | --- | --- |
| STR | *rpsL* | K43R  K88R  Others | 48.9 (44/90)  14.4 (13/90)  18.9 (17/90) |
|  | *gidB* | Frameshift | 17.8 (16/90) |
| INH | *katG* | S315T | 78.2 (104/133) |
|  | *fabG1* | c-15t | 12.8 (17/133) |
|  | *katG+fabG1* | S315T+c-15t | 9.0 (12/133) |
| RIF | *rpoB1* | S450L/F/W | 51.3 (79/154) |
|  |  | H445D/Y/G/L/R/N/Q | 18.9 (29/154) |
|  |  | D435V/F/Y/E | 9.7 (15/154) |
|  |  | L430P/R | 9.7 (15/154) |
|  |  | Others | 10.4 (16/154) |
| EMB | *embB* | M306V/I/L | 71.8 (51/71) |
|  |  | G406D/S | 12.8 (9/71) |
|  |  | Q497R/K | 9.8 (7/71) |
|  |  | Others (D354A, Y319S) | 5.6 (4/71) |
| FQs | *gyrA* | D94G/A/Y/N | 45.5 (8+6+1 (15/33) |
|  |  | A90V | 36.4 (12/33) |
|  |  | D89N | 6.0 (2/33) |
|  | *gyrB* | E501D/V | 12.1 (4/33) |
| AMGs | *rrs1* | a1401g | 100 (1/1) |
| ETH | *ethA* | frameshift | 20 (9/45) |
|  |  | Others | 15.6 (7/45) |
|  | *fabG1* | c-15t | 64.4 (29/45) |
| PZA | *pncA* | frameshift | 21.7 (10/46) |
|  |  | G105D | 13.6 (6/46) |
|  |  | L4S | 13.6 (6/46) |
|  |  | others | 52.1 (24/46) |
